# Editorial: Nematophagous fungi as nematode control agents

**DOI:** 10.3389/ffunb.2023.1353132

**Published:** 2024-01-17

**Authors:** Filippe Elias de Freitas Soares, Liliana Aguilar-Marcelino, Fabio Ribeiro Braga

**Affiliations:** ^1^Laboratório de Biotecnologia e Bioquímica Aplicada, Departamento de Química, Universidade Federal de Lavras, Lavras, Brazil; ^2^Centro Nacional de Investigación Disciplinaria en Salud Animal e Inocuidad, Instituto Nacional de Investigaciones Forestales, Agrícolas y Pecuarias (INIFAP), Jiutepec, Morelos, Mexico; ^3^Laboratório de Parasitologia Experimental e Controle Biológico, Universidade Vila Velha, Espírito Santo, Vila Velha, Brazil

**Keywords:** nematophagous fungi, nematode, biological control, metabolites, nematicidal

Inappropriate and excessive use of chemicals leads to several negative effects on One Health. Therefore, research on alternative measures of pest control is urgent and necessary. Moreover, the United Nations 2030 agenda emphasizes the objective of achieving food security and promoting sustainable agriculture. Thus, the use of biological control is highly necessary. In this context, microbial control using fungi stands out. Some specific fungi are natural enemies of nematodes, as the fungi consume the nematodes. These carnivorous fungi are known as nematophagous fungi (NF).

NF are present in almost all taxonomic groups of the Fungi kingdom and can be classified into five groups: nematode-trapping/predators, opportunistic or ovicidal, endoparasites, toxin-producing fungi, and producers of special attack devices (Soares et al., 2018).

Those microorganisms have biotechnological interests, beyond biological control. Furthermore, the production of enzymes and nanoparticles derived from these organisms that have proven nematicidal activity is highlighted (Barbosa et al., 2019; Soares et al., 2023). Thus, in this Research Topic, Al-Ani et al. reviewed the role of NF in biotechnology and sustainable agriculture. They divided NF into two types according to the mechanisms that affect nematodes: direct (ectoparasites, endoparasites, cyst, or egg parasites producing toxins, and producers of special attack devices) or indirect effects (paralyzing toxins that affect the life cycle of nematodes). In addition, the authors discussed some molecular mechanisms of NF regarding the adaptation of NF to the environment and their action on nematodes.

One of the most prominent NF as a product and in research for the control of parasitic nematodes of plants of interest is *Pochonia chlamydosporia*. This opportunistic fungus has the ability to grow on chitosan as its only nutrient source. Chitosan is a polysaccharide generated from the N-deacetylated form of chitin. In addition, it is effective in the control of plant pests and diseases. In this Research Topic, Lopez-Nuñes et al. discuss the beneficial endophytic action that *P. chlamydosporia* performs on plants and how the combined use of chitosan and nematophagous fungi could be a novel strategy for the biological control of nematodes and other root pathogens.

Still regarding *P. chlamydosporia*, Ciancio et al. developed two models to simulate the interactions between the fungus and the root-knot nematode *Meloidogyne incognita* in a rhizosphere microcosm. Using complex models, these authors sought to describe the biocontrol interaction between *P. chlamydosporia* and *M. incognita*. Thus, they observed that non-parasitic fungal behavior affects biocontrol efficacy by a further density-independent host regulation mechanism.

On the other hand, for adequate production of NF, several parameters must be evaluated and optimized. Therefore, aspects of fermentation, formulation, predation, and half-life are important. In this Research Topic, Courtine et al. evaluated, over the course of two decades of laboratory culture, a strain of *Drechmeria coniospora*. They observed that, after that period, *D. coniospora* became more virulent during its infection of the nematode model *Caenorhabditis elegans*. They noticed that compared to the original isolate (Swe1), the cryo-archived strain (Swe3) was faster in killing the nematode. They suggested that a small number of non-synonymous mutations in protein-coding genes were responsible for such action.

## Author’s note

Fabio Ribeiro Braga: Scholarship CNPq.

## Author contributions

FS: Writing – original draft, Writing – review & editing. LA-M: Writing – review & editing. FB: Writing – review & editing.
